# Asymmetric Underlying Mechanisms of Relation-Based and Property-Based Noun–Noun Conceptual Combination

**DOI:** 10.3389/fpsyg.2021.567971

**Published:** 2021-07-26

**Authors:** Mingyeong Choi, Sangsuk Yoon

**Affiliations:** ^1^Institute of Social Science Research, Pusan National University, Busan, South Korea; ^2^Department of Marketing, University of Dayton, Dayton, OH, United States

**Keywords:** combinatorial semantics, semantic integration, intrinsic and extrinsic semantic features, taxonomic and thematic relations, semantic memory

## Abstract

Conceptual combination is a fundamental human cognitive ability by which people can experience infinite thinking by artfully combining finite knowledge. For example, one can instantly combine “cactus” and “fish” together as “prickly fish” even if one has never previously heard of a “cactus fish.” Although two major combinatorial types—property and relational combinations—have been identified, the underlying processes of each remain elusive. This study investigates the asymmetric processing mechanisms underlying property and relational combinations by examining differential semantic activation during noun–noun conceptual combination. Across two experiments utilizing each combinatorial process as semantic priming and implementing a lexical decision task immediately after combination, we measure and compare the semantic activation patterns of intrinsic and extrinsic semantic features in these two combinatorial types. We found converging evidence that property and relational combinations involve asymmetric semantic information and entail distinct processing mechanisms. In property combination, the intrinsic feature in the modifier concept showed greater activation than the semantic feature of the same dimension in the head concept. In contrast, in relational combination, the extrinsic semantic feature in the head concept and the whole modifier concept showed similar levels of activation. Moreover, our findings also showed that these patterns of semantic activation occurred only when the combinatorial process was complete, indicating that accessing the same lexical-semantic information is not sufficient to observe asymmetric patterns. These findings demonstrate that property combination involves replacing a specific semantic feature of the head noun with that of the modifier noun, whereas relational combination involves completing the semantic feature of the head noun with the whole modifier concept. We discuss the implications of these findings, research limitations, and future research directions.

## Introduction

How would you interpret the following noun–noun combinations: “feather luggage” or “piano blanket?” The purpose of this study was to investigate the processing mechanisms underlying property and relational combinations. To this end, we examined the differential semantic activations that occur when faced with various noun–noun conceptual combinations. Conceptual combination is a process of integrating multiple semantic concepts into a novel coherent representation. To semantically integrate multiple concepts, one should first access the semantic information of those concepts and determine how they fit together to form a novel one. This is an essential and fundamental human cognitive ability by which people can create unlimited novel concepts from a limited set of existing ones. In this regard, conceptual combination is regarded as a cornerstone that can shed light on human creativity (Murphy, 2004; Prinz, 2004).

Conceptual combination has been studied mainly in the noun–noun form (the former noun is the modifier, and the latter is the head) since it better represents the combinatorial coordination processes than other simpler combinations, such as adjective–noun combinations (Wisniewski, 1997). While there are theoretically infinite possible ways to combine two constituent nouns to create new meanings, previous studies have proposed two major types: *relational* combination and *property* combination. In relational combination, the two constituent concepts play independent and complementary thematic roles; in contrast, in property combination, one constituent concept is diminished to a specific semantic feature. For example, one can interpret “cactus fish” in a relational combination (e.g., “fish that eat cactus”) where both the modifier (“cactus”) and head concepts (“fish”) remain as individual concepts in the interpretation. However, one might also interpret “cactus fish” as a property combination (e.g., “prickly fish”) where the modifier concept (“cactus”) is diminished to a value (“prickly”) of a specific dimension (“texture”) and replaces the value of the same dimension of the head concept (“slithery” of “fish”).

Although how to categorize specific verbal interpretations from noun–noun conceptual combinations into a relational or property combination has been well defined, relatively little work has examined the underlying processing mechanisms. Moreover, previous models of conceptual combination were developed independently with few direct comparisons between the two combinatorial processes. In the current research, we present a novel approach for examining the asymmetric combinatorial processing of property and relational combination. Across two experiments using each type of combination as semantic priming, we measure and compare the semantic activation patterns of the two different types of semantic features (intrinsic or taxonomic vs. extrinsic or thematic) in property and relational combinations.

### Relation-Based and Feature-Based Models of Conceptual Combination

Cognitive models of noun–noun conceptual combination can be subcategorized into two types: a relation-based or feature-based approach (Gagné, 2000; Choi and Shin, 2010). The relation-based approach posits that conceptual combination is the processes and consequences of selecting an appropriate thematic relation between the head and modifier concepts. Thematic relations indicate “the external or complementary relations among objects, events, people, and other entities that co-occur or interact together in space and time (Lin and Murphy, 2001, p. 3).” For example, the thematic relation of “location” can be used to link “mountain cloud” and result in the following interpretation: “cloud LOCATED in a mountain.” Ten to fifteen largely overlapping thematic relations have been identified across different research, including the thematic relations of “about” (e.g., “mountain magazine”), “make” (e.g., “milk cow”), and so on (Downing, 1977; Levi, 1978; Gagné and Shoben, 1997). The competition among relations in nominals (CARIN) model (Gagné and Shoben, 1997; Gagné and Spalding, 2009), a pivotal model of the relation-based approach, provides a theoretical framework regarding how an appropriate thematic relation is selected to link two constituent concepts. The CARIN model mainly focuses on explaining relational combination and suggests that relational combination is preferentially attempted (i.e., the primacy of relational combination). The model argues that other types of combinatorial processes—including property combination—occur when relational combination attempts fail.

On the other hand, the feature-based approach assumes that the human knowledge system is grounded on a scheme structure in which conceptual knowledge consists of multiple dimensions with unique values. For example, “elephant” is composed of multiple feature dimensions with a unique value for each one, such as “gray” for the COLOR dimension and “big” for the SIZE dimension. In this regard, the feature-based approach explains conceptual combination as a slot-filling process (Smith and Medin, 1981; Murphy, 1990; Choi and Shin, 2013; Rumelhart, 2019). For example, “elephant ant” can be interpreted as “big ant” as the value of the SIZE dimension of the head noun, “ant” (i.e., “small”), is replaced or filled with the value of the same dimension of the modifier noun, “elephant” (i.e., “big”). Most cognitive models of noun–noun conceptual combination, such as the dual-process model (Wisniewski, 1996; Wisniewski and Love, 1998), interactive property attribution (IPA) model (Estes and Glucksberg, 2000), and the total-partial (TP) model (Choi et al., 2007), have elucidated how the value of a certain dimension of the head concept is systematically modified by the modifier concept throughout the shared dimension between the two constituent concepts. These models mainly focus on falsifying the primacy of relational combination proposed by the CARIN model and elucidating the process of property combination. For example, the dual-process model demonstrates that when there is a high similarity between the modifier and head nouns, property combination occurs faster than relational combination, which is in contrast with the primacy of relational combination (Wisniewski and Love, 1998). In addition, the IPA model shows that high saliency in the modifier noun leads to a property combination rather than a relational combination (Estes and Glucksberg, 2000).

Relation- and feature-based models theorize how and when property or relational combination occurs and focus more on explaining one of these two combinatorial types. However, relatively few studies have attempted to directly compare the distinct underlying processes of property and relational combinations. Elaborating the characteristics of only one combination type is insufficient to understand how people combine concepts and expand their knowledge. Thus, here we attempt to compare the underlying mechanisms of the two combinatorial processes directly. By directly comparing the semantic information and process mechanism in property and relational combinations, we expect to achieve more comprehensive understanding of conceptual combination.

### Role of Intrinsic and Extrinsic Features in Property and Relational Combinations

Among many studies that classify semantic information into distinct types, extensive discussions on the discrepancy between taxonomic and thematic systems have been published (Lin and Murphy, 2001; Gentner and Kurtz, 2006; Estes et al., 2011; Mirman et al., 2011, 2017). Taxonomic relations refer to a hierarchical semantic system in which concepts are classified into different levels with varying specificity (e.g., animal—mammal—dog—golden retriever—my blond dog). Taxonomic classifications are based on shared features between concepts, and these features are generally perceptual (e.g., furry, four legs, and viviparous). In the history of semantic system research, the taxonomic system has been the core and basis of human semantic organization.

However, subsequent research has revealed that taxonomic relations cannot fully capture the entire spectrum and richness of semantic memory, and the thematic system has a unique and significant contribution that complements the long-standing taxonomic knowledge system (Kalénine et al., 2009, 2012; Mirman and Graziano, 2012a,b). Thematic relations indicate external or complementary relations among objects, events, people, and other entities that co-occur or interact together in space and time (e.g., dog–leash and doctor–hospital). For example, even though “dog” and “leash” do not share any perceptual or conceptual feature, they are closely related by the theme, “walking,” fulfilling complementary roles in this context (e.g., a leash is used to take a dog for a walk).

Recent neuroimaging studies have revealed supporting evidence that these two semantic systems are dissociated both functionally and anatomically in the brain. Studies on neuroanatomical dissociation for taxonomic and thematic knowledge in the brain have shown that people show increased activation in the anterior temporal lobe (ATL) when they process taxonomic (feature-based) knowledge. In contrast, the temporoparietal junction (TPJ) and angular gyrus (AG) show increased activation when people engage in the process of thematic knowledge (Sachs et al., 2008; Schwartz et al., 2011; Lewis et al., 2015).

Among similar discussions on semantic systems, the study by Barr and Caplan (1987) is particularly worth mentioning. They classified semantic features into two distinct types: intrinsic and extrinsic. Intrinsic features are semantic features that can exist independently without additional entities, whereas extrinsic features express relations between entities or events that cannot exist alone and need additional entities to be completely processed and understood. For example, among the various semantic features of “knife,” “sharp” is an intrinsic feature because one can understand what it means without any additional information, whereas “to cut” is an extrinsic feature since it needs another entity, such as an object that would be cut by the knife, to be understood.

Although discussions on the disparities between taxonomic versus thematic systems and intrinsic versus extrinsic features have been developed separately, they share similarities with regard to semantic information. Intrinsic or taxonomic semantic features refer to semantic information that does not need any other entities to be semantically complete and focuses on describing what an entity has as its feature considered in isolation. For example, among the semantic features of “hammer,” “solid” is an intrinsic/taxonomic semantic feature because “solid” itself does not need any other entities to be understood as “solid.” On the other hand, extrinsic or thematic semantic features refer to semantic features that need other entities to complete semantic processes. They emphasize the interaction between entities or concepts. For example, among the semantic features of “hammer,” “to hit” is an example of an extrinsic/thematic semantic feature because “to hit” needs another entity that would be hit by the hammer to be understood (Barr and Caplan, 1987).

These two types of semantic features are asymmetrically involved in property and relational combinations. Choi et al. (2007) first related intrinsic semantic information to property combination and extrinsic semantic information to relational combination. The authors showed that property combination was facilitated when the modifier noun had salient intrinsic features and when the head noun had corresponding or relevant feature dimensions to the salient intrinsic features of the modifier noun. On the other hand, relational combination was facilitated when the head noun had salient extrinsic features and when the modifier noun was relevant to the salient extrinsic features of the head noun. For instance, “feather blanket” was rapidly interpreted as “light blanket” (i.e., property combination) because the salient intrinsic feature of the modifier, “feather” (e.g., “light”), could modify the value of the relevant feature dimension (WEIGHT dimension) of the head. However, although “feather soup” has the same modifier, it was not easily interpreted as a property combination because the relevant feature dimension (WEIGHT dimension) was relatively more difficult to find in the head noun, “soup.” On the other hand, “dish blanket” was rapidly interpreted as “a blanket that covers dishes” (i.e., relational combination) because the salient extrinsic feature of the head, “blanket” (i.e., “to cover”), can take the modifier, “dish,” as a semantically congruent object to be covered. Although “oxygen blanket” has the same head noun, “blanket,” it was not easily interpreted as a relational combination because the modifier, “oxygen,” was less plausible to be covered by a blanket.

They also highlighted why salient intrinsic features should be in the modifier concept, while salient extrinsic features should be in the head concept in their model. In noun–noun conceptual combination, each constituent concept plays a different role in semantic integration (Costello and Keane, 2000; Estes and Glucksberg, 2000; Keane and Costello, 2001). The noun–noun form of compound words naturally gives the former noun the modifier role with the latter being the focal concept. For example, “cactus (modifier) fish (head)” is about a kind of “fish” and not about a kind of “cactus.” Thus, the head noun contributes the most information to the compound. This nature of the head noun corresponds well to the characteristics of extrinsic features in that it needs something to be modified to be understood. On the other hand, the role of the modifier noun is limited to modifying a particular aspect of the focal concept, and this nature of the modifier noun corresponds to the characteristics of the intrinsic semantic features because it pertains to a specific semantic feature that can modify the head without requiring other entities or concepts.

Boylan et al. (2017) investigated the neural mechanisms underlying property and relational combinations using functional magnetic resonance imaging (fMRI). They also showed converging evidence that taxonomic and thematic semantic features are asymmetrically involved in property and relational combinations. Property combination is associated with the ATL where taxonomic knowledge is stored and processed, whereas relational combination is associated with the AG where thematic knowledge is stored and processed. These findings support the idea that taxonomic (intrinsic) features are more involved in property combination, while thematic (extrinsic) features are involved more in relational combination.

To directly compare the underlying online mechanisms of property and relational combinations, we leverage the prediction that different types of semantic features asymmetrically influence the processing of property and relational combinations. Property combination specifically involves intrinsic semantic features, whereas relational combination involves extrinsic semantic features. Moreover, we decided to use the terms “intrinsic” and “extrinsic” rather than “taxonomic” and “thematic” to designate the semantic features associated with property and relational combinations, respectively. The definitions of intrinsic and extrinsic semantic features more directly describe semantic properties itself rather than referring to the relationship between elements in each semantic system as in taxonomic and thematic systems.

### Asymmetric Processing Mechanisms of Property and Relational Combinations

One of the caveats of previous studies on noun–noun conceptual combination is that classifications of property and relational combinations are based on the unique syntactic features expressed in the verbal outcomes of interpretation generation tasks (Wisniewski, 1996; Costello and Keane, 1997; Wisniewski and Love, 1998; Estes, 2003). Thus, while how to categorize generated interpretations into either a property or relational interpretation has been well established, the underlying processes of these two interpretations remain elusive. For two noun concepts to be semantically integrated, semantic knowledge of both constituent concepts should be activated. Then, the activated semantic knowledge should be coherently reconstructed through a series of coordinated modifications to seek an appropriate meaning for the novel compounds (Swinney et al., 2007). Thus, specifying which semantic knowledge is used in the final interpretations is insufficient to fully delineate the entire processes of semantic integration. It is important to precisely elucidate the detailed course and mechanism of semantic combinatorial processing, determine which type of semantic information is involved, assess what course of operation is applied to the information, and most importantly, describe how distinct the information and processes are between property and relational combinations.

However, there are relatively few studies on how specific types of semantic features are activated and utilized during combinations. As shown previously, Choi et al. (2007) proposed that property combination entails the replacement of an intrinsic feature of the head noun with that of the modifier noun, whereas relational combination involves matching an extrinsic feature of the head noun with the entire modifier concept. For example, if “feather luggage” is interpreted as “light luggage,” (i.e., a property interpretation), this could occur through feature replacement in which the value of the shared dimension (WEIGHT dimension) of the head (i.e., “heavy” of “luggage”) is replaced with the value of the shared dimension of the modifier noun (i.e., “light” of “feather”). On the other hand, an example of a relational interpretation would be if “piano blanket” is interpreted as “a blanket to cover the piano” where an extrinsic feature of the head noun (i.e., “to cover something” of “blanket”) matches the whole modifier concept (i.e., “piano”). The authors also showed supporting empirical evidence that noun–noun pairs that have a salient intrinsic feature in the modifier noun facilitated property combination, whereas noun–noun pairs that have salient extrinsic features in the head noun facilitated relational combination. However, the study was also based on generated verbal interpretations rather than explicit examination of whether a particular intrinsic or extrinsic semantic feature was activated and exploited during the course of combination.

In the current study, taking the idea proposed by Choi et al. (2007) one step further from, we attempt to directly examine whether a salient intrinsic feature of the modifier noun replaces a feature of the same dimension of the head noun in property combination and whether a salient extrinsic feature of the head noun is linked with the whole concept of the modifier noun. To test this idea, we employ each type of combination as semantic priming and measure the semantic activation patterns of intrinsic and extrinsic semantic features using a lexical decision task (LDT) immediately after finishing combination processes. If a salient feature of the modifier noun replaces a feature of the same dimension of the head noun in property combination, the response time (RT) on the LDT for the intrinsic feature of the modifier noun will be shorter than that for a semantic feature in the same dimension for the head noun. For example, if one interprets “feather luggage” as “light luggage” [i.e., the value of the WEIGHT dimension of the head noun (“heavy” of “luggage”) is replaced with a feature of the modifier noun (“light” of “feather”)], we will find a faster RT for “light” and a slower RT for “heavy” on the LDT immediately after completing this property combination. On the other hand, if a salient extrinsic feature of the head noun links to the whole modifier noun in relational combination, the RT for the whole modifier and that for the extrinsic feature of the head noun will not be different. For example, if one interprets “piano blanket” as “a blanket that covers the piano” [i.e., the entire modifier (“piano”) is successfully coupled with an extrinsic feature of the head noun (“cover”)], the RT for “cover” and that for “piano” will not be different. In Experiment 1, for property combination, we contrast the RT on the LDT for intrinsic semantic features of the modifier nouns and that for a semantic feature of the same dimension for the head noun, whereas for relational combination, we compare the RT on the LDT for extrinsic features of the head nouns with that for the whole concept of the modifier nouns. Experiment 2 examines whether the asymmetric RT patterns observed in Experiment 1 can be attributed to distinctive combinatorial processes between property and relational combinations or to mere lexical-semantic processes of constituent concepts.

## Experiment 1

Experiment 1 aims to directly compare the combinatorial processes of property and relational combinations using a semantic priming paradigm and a subsequent LDT. For the LDT, we selected a probe word for each modifier noun (modifier-related probe) and head noun (head-related probe) that enabled us to investigate whether the feature of the modifier noun or of the head noun was actually activated as a result of property and relational combinations.

### Materials and Methods

#### Participants

Two hundred participants in total (110 females; mean age = 38.59 years, *SD* = 13.70, range = 18–82) were recruited from the Amazon Mechanical Turk panel in exchange for a cash payment with restrictions that their geographical location was in the United States and English was their primary language. We obtained informed consent from all participants according to a protocol approved by the Institutional Review Board of the institute to which one of the authors is affiliated.

#### Experimental Stimuli

We created 40 novel noun–noun word compounds that are more prone to be interpreted either as property combination (property compound; e.g., “feather luggage”) or as relational combination (relational compound; e.g., “piano blanket”). Then, we created modifier and head probe word for each compound. The full list of stimuli used in Experiment 1 including property/relational compounds and modifier/head probes are available at OSF (please refer to the data availability statement).

##### Noun–Noun Compound Words

First, we created 45 noun–noun compounds (22 for property and 23 for relational compounds) using concrete English nouns following the criteria described by Choi et al. (2007). Employing the same criteria, we systematically selected the modifier and head nouns. For property compounds, we selected modifier nouns that had at least one salient intrinsic feature (e.g., “light” of “feather” or “red” of “blood”) and head nouns that shared the same feature dimension of the salient intrinsic feature of the modifier noun. For relational compounds, we selected head nouns that had at least one salient extrinsic feature (e.g., “to cover” of “blanket” or “to cut” of “scissors”) that semantically matched the modifier concept.

To ensure the interpretation bias of each compound, we recruited 20 participants from Amazon Mechanical Turk [7 females; mean age = 35.3 years, standard deviation (*SD*) = 8.98 years] in exchange for a cash payment and asked them how they interpreted novel compounds. The participants who misunderstood the task and generated invalid responses (e.g., nonwords, random numbers, and random letters) were excluded prior to the analysis (*n* = 6). The generated interpretations were categorized as one of the four interpretation types (property, relational, hybrid, and exocentric interpretation) defined in previous research (Wisniewski, 1996; Wisniewski and Love, 1998; Costello et al., 2004; Choi et al., 2007). Although a novel noun–noun compound can have variability in its interpretation, property and relational combinations are the most common interpretations, while other interpretation types are rare, occupying less than 10% of the total responses (Choi et al., 2007). We excluded these minor types of interpretations and only used the compounds that were interpreted in the corresponding manner (i.e., property interpretation for property compounds and relational interpretation for relational compounds) for over 50% of the total interpretations (excluded 3 compounds). In addition, we excluded the compounds that showed more frequent reversed interpretations (e.g., more relational interpretations for property compounds and vice versa; excluded 2 compounds). Finally, these criteria confirmed a total of 40 novel compounds including 20 for each compound type. The interpretation bias analysis confirmed that the property compounds had property interpretations and that the relational compounds had relational interpretations as the majority (mean % of property interpretations for the property compounds = 92.8%, *SD* = 8.57%, range = 72.7–100% vs. mean % of relational interpretations for the relational compounds = 92.5%, *SD* = 10.32%, range = 66.7–100%; see Table 1 for details). As shown in Table 2, we also collected plausibility and familiarity ratings from an independent sample using a 5-point scale and confirmed that there was no significant difference in plausibility or familiarity between the property and relational compounds [*n* = 200; *t*(38)_plausibility_ = −1.440, *p* = 0.158; *t*(38)_familiarity_ = −0.461, *p* = 0.647]. The linguistic features of property and relational compounds were matched for summed values for letter length, syllabic number, word frequency (Brysbaert and New, 2009), orthographic neighborhood density (Medler and Binder, 2005), and word association (latent semantic analysis over general reading at first year college level; Laham, 1998; Landauer et al., 1998; all dependent sample *t*-test, *p* > 0.6).

**Table 1:** Interpretation bias of property and relational compounds in Experiment 1 and 2.

Property compound words	% of property interpretation	Relational compound words	% of relational interpretation
Blood sky	100.00	Chicken axe	81.82
Butter grip	91.67	Glasses bandage	81.82
Cactus fish	100.00	Piano blanket	66.67
Candy coffee	100.00	Paint blender	100.00
Cherry banana	100.00	Sofa broom	100.00
Dwarf bear	100.00	House crayon	72.73
Elephant ant	100.00	Vase glue	100.00
Feather backpack	85.71	Walnut hammer	100.00
Giraffe kid	90.91	Pizza heater	81.82
Ice soup	87.50	Deer jail	100.00
Pepper cucumber	90.00	Cod knife	100.00
Rainbow chocolate	100.00	Guacamole ladle	91.67
Rubber wood	83.33	Alley marathon	100.00
Sandpaper skin	100.00	Bagel oven	90.91
Skunk perfume	91.67	Rose painter	90.91
Snow crow	78.57	Spider pesticide	100.00
Steel glass	83.33	Flower scissor	92.31
Sugar lemon	72.73	Cake spatula	100.00
Volcano candle	100.00	Mug towel	100.00
Zebra ladybug	100.00	Violin warehouse	100.00
Average	**92.77**	Average	**92.53**

**Table 2:** Means (standard deviations) of semantic and linguistic features of the compound and probe words in Experiments 1 and 2.

	Compounds							Modifier probe					Head probe				
	PLA	FAM	LSA	Sum. LEN	Sum. SYL	Sum. WF	Sum. OND	VAL	LEN	SYL	WF	OND	VAL	LEN	SYL	WF	OND
Property compound	3.13 (0.73)	1.40 (0.68)	0.16 (0.12)	11.10 (2.29)	3.50 (1.15)	70.61 (86.36)	7.30 (5.73)	4.75 (0.55)	5.80 (1.70)	1.65 (0.81)	85.43 (154.63)	5.15 (5.58)	4.10 (0.79)	5.45 (1.57)	1.70 (0.73)	112.07 (318.88)	4.50 (4.83)
Relational compound	3.60 (1.19)	1.65 (0.86)	0.09 (0.11)	11.20 (2.35)	3.60 (1.10)	55.12 (110.14)	7.50 (6.65)	4.25 (0.71)	5.45 (1.43)	1.60 (0.82)	19.40 (30.32)	5.75 (6.22)	4.68 (0.52)	4.75 (1.62)	1.35 (0.49)	82.43 (133.29)	6.90 (6.34)

*PLA, plausibility rating (max = 5); FAM, familiarity rating (max = 5); LSA, latent semantic association; Sum. LEN, summed letter length; Sum. SYL, summed syllabic number; Sum.WF, summed word frequency; Sum. OND, summed orthographic neighborhood density; VAL, cue validity rating (max = 5); LEN, letter length; SYL, syllabic number; WF, word frequency; and OND, orthographic neighborhood density*.

##### Probe Words

Each noun–noun compound had two probe words; one modifier-related word (modifier probe) and one head-related word (head probe). Since the property compounds were initially constructed around the most salient intrinsic features of the modifier word, the modifier probes were the adjective form of these intrinsic features. The head probe was also the adjective form of the semantic feature that corresponded to the feature dimension of the modifier probe. The saliency of the selected intrinsic and extrinsic features was expected to make them easier to be detected and thus be utilized in the combinatorial processes, enabling us to better examine the hypothesis. In the example “cherry swan” (the highly probable interpretation is “red swan”), the modifier probe is “red” (a salient feature of “cherry”), and the head probe is “white” [the value of the same dimension as “red” (COLOR dimension) in “swan”]. If a feature of the head noun is replaced with a feature of the modifier noun, we can expect to observe a slower RT for the head probe (“white”) than for the modifier probe (“red”) on the LDT following the property combinatorial process.

In creating novel relational compounds, we first selected head nouns that had salient extrinsic features (e.g., “hammer”—“hit”) and then matched a modifier concept (e.g., “walnut”) with which the extrinsic feature of the head noun would be semantically in accordance. Thus, the head probe was the transitive verb form of the salient extrinsic feature of the head concept, which would lead to the relational combination. The modifier probe was the noun form of a specific part of the modifier concept that could play a role as an object to the transitive verb of the head probe. For example, in “piano blanket” (the highly probable interpretation is “a blanket that covers a piano”), the head probe is “cover,” and the modifier probe is the “keyboard” that the blanket covers. This enables us to examine whether the relational combination is completed by semantically matching the extrinsic feature of the head concept with the whole modifier concept, which means inserting the modifier concept into the object position of the transitive verb of the extrinsic feature of the head concept. In selecting a modifier probe, we chose a noun to designate the part of the modifier that has an essential role in the combination rather than using the original and thus redundant modifier word (“keyboard” vs. “piano”) to avoid the repetition priming. This method aims to examine whether the relational combination involves the entire modifier concept, rather than a specific feature of the concept. If the relational combination is completed by inserting the whole modifier concept into the slot of the extrinsic feature of the head concept, the semantic activations of the head and modifier probes will be the same, resulting in a nonsignificant difference in the RT on the LDT preceded by the relational combinatorial process.

In a separate test (*n* = 601, female = 326; mean age = 39.70 years, *SD* = 12.74), we collected ratings of how representative each probe was for each constituent concept (e.g., “How well does ‘red’ represent ‘cherry’?”) using a 5-point scale to confirm the validity of the probe words. This ensured that the median ratings for each probe were all greater than three (all one-sample *t*-tests; *t*s > 6.00, *p*s < 0.001). The linguistic features of all the probe words including the length, syllabic number, frequency, and orthogonal neighborhood density were controlled across all conditions (all independent sample *t*-test; *p* > 0.05; see Table 2 for details).

#### Experimental Design and Task

**Figure 1:**
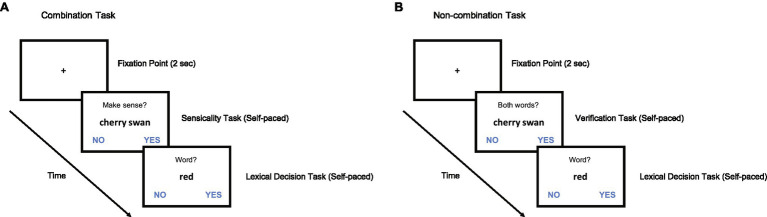
An example trial in Experiment 1 and 2. (**A**) Combination task condition in Experiments 1 and 2: Participants viewed novel noun–noun compound words and decided whether the given compound was meaningful (sensicality task) followed by the lexical decision task (LDT). (**B**) Non-combination task condition in Experiment 2: Participants viewed novel noun-noun compound words and decided whether both the modifier and head nouns were words (verification task) followed by LDT.

We used a 2 (combinatorial type: property vs. relational; between-subjects) × 2 (probe type: modifier vs. head; within-subjects) mixed design. Participants were randomly assigned either to property or relational combinatorial type conditions. In each trial, participants were asked to make two judgments. First, they were presented with a novel noun–noun compound and asked to judge whether the given compound made sense (yes or no; sensicality task). If participants could create any sensible interpretation, they would press the ‘yes’ button. However, if they failed to create any sensible interpretation, their answer would be ‘no.’ Immediately after the sensicality judgment, participants were given a series of letters and asked to make a judgment regarding whether the given letters indicated a word (yes or no; LDT). Among the target trials (i.e., trials where the given letters indicated a word), half of the words were modifier probes, while the other half were head probes. Each participant completed five practice trials and 60 main trials that consisted of 10 head and modifier trials each and 40 filler trials (nonsensical or nonword trials). All trials were fully randomized, and no words were repeated. The accuracies and latencies of both tasks were measured (schematic descriptions of the experimental procedure are shown in Figure 1).

### Results

Prior to the data analysis, we excluded invalid responses with the following rules: (1) trials where participants failed to provide correct answers on the LDT (4%); (2) trials where participants could not come up with a meaning for a given noun–noun compound (i.e., ‘no’ answers on the sensicality task; 22.28%) since we were interested in semantic activations induced by combinatorial processes; (3) RTs on the LDT that were longer than 1.50 s or shorter than 0.15 s (6.2%; we applied these RT outlier criteria from Wittenbrink et al., 1997; Sechrist and Stangor, 2001; Crisp and Nicel, 2004; West et al., 2014); (4) RTs on the sensicality task that were shorter than 0.15 s (0.05%); and (5) participants who had less than 50% valid responses from the preceding exclusion criteria (4.6%). This resulted in the exclusion of 1,483 trials in total (37%).

**Figure 2:**
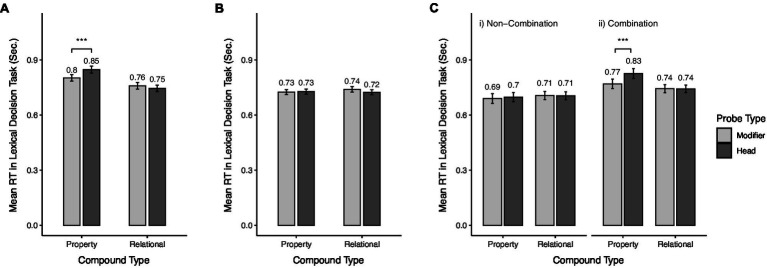
Mean response time (RT) in the lexical decision task (LDT) by conditions in Experiment 1 and 2. **(A)** Mean RT in the LDT by combinatorial type and probe type in Experiment 1, **(B)** Mean RT of the baseline condition in the LDT by combinatorial type and probe type in Experiment 1, and **(C)** Mean RT in the LDT by task type, combinatorial type, and probe type in Experiment 2. Error bars indicate standard errors. ^***^p < 0.001.

For the analysis, we ran a 2 (combinatorial type: property vs. relational; between-subjects) × 2 (probe type: modifier vs. head; within-subjects) mixed ANOVA. We found a significant interaction effect between the combinatorial type and the probe type [*F*(1, 144) = 11.57, *p* < 0.001, $\eta_{p}^{2}$ = 0.07] and a significant main effect for the combinatorial type [*F*(1, 144) = 9.04, *p* = 0.003, $\eta_{p}^{2}$ = 0.06; Figure 2A]. The main effect of probe type was marginally significant [*F*(1, 144) = 3.21, *p* = 0.075, $\eta_{p}^{2}$ = 0.02]. Further contrast results with Tukey’s multiple comparison corrections revealed that in the property combination condition, participants responded slower to head probes than to modifier probes [*M*_head_ = 0.85 s, *SD* = 0.16 vs. *M*_modifier_ = 0.80 s, *SD* = 0.15; *t* = 3.67, *p* < 0.001], whereas they did not show a difference in decision latencies between the head and modifier probes in the relational combination condition [*M*_head_ = 0.75 s, *SD* = 0.15 vs. *M*_modifier_ = 0.76 s, *SD* = 0.16; *t* = −1.14, *p* = 0.257].

Considering the variance across all trials within each subject, we conducted an additional analysis using all valid trials. We regressed the RT on combinatorial type interacting with probe type using a multilevel linear regression with subject-level random intercepts. The results showed that there was a significant combinatorial type × probe type two-way interaction (*b* = 0.06, *SE* = 0.02, *t* = 3.96, *p* < 0.001). Further contrast results showed that participants showed faster RTs for the modifier probes than for the head probes in the property combination trials (*b* = 0.04, *SE* = 0.01, *z* = 3.91, *p* = 0.001). However, the difference in RTs between the head and modifier probes in the relational combination trials was marginally significant (*b* = −0.02, *SE* = 0.01, *z* = −1.73, *p* = 0.084).

Moreover, to rule out potential confounding effects of different parts of speech of the probe words on RTs, we conducted an additional LDT study with a new sample (*N* = 201, female = 87; mean age = 39.16 years, *SD* = 12.73; after excluding outliers, *N* = 169). In the study, participants were shown a constituent word (e.g., “cherry”) as a semantic prime followed by its probe word (e.g., “red”). In this way, we measured the baseline RT of each probe word using a corresponding constituent concept as a prime using a 2 (probe type: head vs. modifier) × 2 (combinatorial type: property vs. relational) within-subjects design. The results of a 2 × 2 repeated-measure ANOVA showed that the main effects of probe type [*F*(1, 168) = 0.826, *p* = 0.365, $\eta_{p}^{2}$ = 0.005] and combinatorial type [*F*(1, 168) = 0.461, *p* = 0.498, $\eta_{p}^{2}=$0.003], and the interaction effect was not significant [*F*(1, 168) = 1.964, *p* = 0.163, $\eta_{p}^{2}=$0.012], indicating that the different parts of speech did not result in differences in RTs (Figure 2B). This finding also indicates that there was no significant difference in lexical decision RTs between property and relational combinations for the modifier and head probes when they were primed by their corresponding constituent concepts.

### Discussion

The asymmetric RTs for the modifier and head probes between property and relational combinations indicate that different semantic features are involved in each type of combination. More importantly, this finding shows that the different semantic features involve differential combinatorial processing mechanisms. In property combination, the faster RTs for modifier probes than for head probes indicate that an intrinsic feature of the modifier noun is activated more than a semantic feature of the head noun for the same dimension. The head probe may also have been relatively inhibited during the process of property combination. This seems to support the idea that property combination is completed by replacing a specific feature of the head with the value of the modifier, resulting in increased activation for a feature of the modifier noun but decreased activation for a feature of the head noun in the same dimension. However, in relational combination, there was no difference in RT between the head and modifier probes, indicating simultaneous activation of the extrinsic feature of the head noun and the whole modifier concept. These results support the idea that relational combination needs both information from the entire modifier concept and the extrinsic feature of the head concept and that feature replacement is not involved, unlike in property combination.

## Experiment 2

The findings of Experiment 1 showed asymmetric activation patterns of the modifier and head probes in the property and relational combinations. However, it is still unclear whether these asymmetric activation patterns resulted solely from differential combinatorial processes or from differential lexical-semantic processing of constituent concepts. We aim to rule out this possibility by comparing semantic activation patterns with and without combinatorial processes as semantic priming. If the asymmetric semantic activation patterns in Experiment 1 arose from the different combinatorial processes of property and relational combinations, these patterns should only appear when participants are asked to create a meaning for a given compound. To examine this, Experiment 2 aims to compare the activation of the modifier and head probes between two task conditions: the combination task condition in which participants are required to integrate the two constituent concepts vs. the non-combination task condition in which participants are required to decide whether or not both modifier and head nouns are words without any semantic integration.

### Materials and Methods

#### Participants

In total, 400 participants (134 females; mean age = 36.07 years, *SD* = 10.25, range = 20–72) were recruited from Amazon Mechanical Turk in exchange for a cash payment.

#### Materials, Experimental Task, and Design

We used a 2 (task type: combination vs. non-combination; between-subjects) × 2 (combinatorial type: property vs. relational; between-subjects) × 2 (probe type: modifier vs. head probe; within-subjects) mixed design. Participants were randomly assigned to one of the four between-subjects experimental conditions. The overall procedures were the same as those for Experiment 1, except that the non-combination task condition required judgment of whether or not both the modifier and head nouns were words (verification task: yes or no) before the LDT, whereas the combination task condition involved judgment of whether the given compound was meaningful (sensicality task: yes or no) before the LDT as in Experiment 1. To verify whether both the nouns of a given compound were words or not, participants had to access semantic information of both constituent nouns, but they did not need to attempt to create new meaning by combining them. However, to create meaning for a novel noun–noun compound, participants needed to implement a combinatorial process in addition to access the semantic information of those nouns. By comparing the RT patterns for the modifier and head modifier probes in the following LDT, we can test whether the asymmetric semantic activation patterns shown in Experiment 1 can be attributed solely to combinatorial processes. The compound and probe stimuli were the same as those in Experiment 1.

### Results

Using the same exclusion criteria as in Experiment 1, we excluded a total of 4,122 responses (approximately 51%) from the data analysis [wrong LDT responses (16.33%)]; wrong answers on the verification task or trials in which participants did not create any meaning for the sensicality task (i.e., ‘no’ responses on the sensicality task in the combination task condition; 14.46%); RTs shorter than 0.15 s or longer than 1.50 s on the LDT (12.55%); RTs shorter than 0.15 s on the sensicality or verification task (1.34%); and respondents who had less than 50% valid trials from all preceding criteria (6.85%).

Consistent with the hypothesis, the activation pattern of the non-combination task condition was different from that of the combination task condition (Figure 2C). A 2 (task type: combination vs. non-combination; between subjects) × 2 (combinatorial type: property vs. relational; between-subject) × 2 (probe type: modifier vs. head probe; within-subjects) mixed ANOVA showed a marginally significant three-way interaction effect [*F*(1, 204) = 3.26, *p* = 0.073, $\eta_{p}^{2}$ = 0.02]. Further contrast results with Tukey’s multiple comparison corrections showed that in the combination task condition, the property compounds showed slower LDT RTs for the head probe than for the modifier probe (*M*_head_ = 0.83 s, *SD* = 0.18 vs. *M*_modifier_ = 0.77 s, *SD* = 0.17; *t* = 3.98, *p* < 0.001), whereas the relational compounds did not show any significant difference in RTs between the head and modifier probes (*M*_head_ = 0.74 s, *SD* = 0.13 vs. *M*_modifier_ = 0.74 s, *SD* = 0.14; *t* = −0.06, *p* = 0.951). This is the same result as in Experiment 1. However, in the non-combination task condition, there was no difference in LDT RTs between the head and modifier probes for either the property (*M*_head_ = 0.70 s, *SD* = 0.19 vs. *M*_modifier_ = 0.69 s, *SD* = 0.20; *t* = 0.59, *p* = 0.557) or relational compound (*M*_head_ = 0.71 s, *SD* = 0.17 vs. *M*_modifier_ = 0.71 s, *SD* = 0.18; *t* = −0.13, *p* = 0.899). Moreover, in the relational compounds, the mean LDT RTs between the combination and non-combination task condition for the modifier (*t* = −1.08, *p* = 0.282) and head probes (*t* = −1.10, *p* = 0.274) were not different.

We also regressed the RT on task type interacting with combinatorial type and probe type using a multilevel linear regression with subject-level random intercepts to consider the variance across all trials within each subject. The results were consistent with the ANOVA results. There was a marginally significant task type × combinatorial type × probe type three-way interaction (*b* = 0.05, *SE* = 0.02, *t* = 1.95, *p* = 0.051). Further contrast results showed that in the combination task condition, participants showed faster LDT RTs for the modifier probes than for the head probes in the property combination trials (*b* = 0.06, *SE* = 0.01, *z* = 4.47, *p* < 0.001), while they showed nonsignificant difference in LDT RTs between the modifier and head probes in the relational combination trials (*b* = −0.0002, *SE* = 0.01, *z* = −0.01, *p* = 0.991). On the other hand, in the non-combination task condition, participants did not show significant differences in RTs between the modifier and head probes both in the property (*b* = 0.01, *SE* = 0.01, *z* = 0.95, *p* = 0.343) and relational combination trials (*b* = 0.0003, *SE* = 0.01, *z* = 0.03, *p* = 0.977).

### Discussion

The results of Experiment 2 demonstrate that the asymmetric semantic activation pattern shown in Experiment 1 occurs only when a combinatorial process is involved (combination task condition) but does not occur when the same semantic information is processed without any combinatorial processes (non-combination task condition). The results corroborate the hypothesis that property combination is a process of replacing a specific semantic feature of the head concept with that of a modifier concept, whereas relational combination is a process of completing the semantic feature of the head concept with the whole modifier concept. Specifically, in the combination task condition for property combination, the results show higher activation of the modifier probe than of the head probe, but no such pattern is present in the non-combination task condition. This indicates that the asymmetric activation pattern of the modifier and head probes was induced from combinatorial processes rather than from the mere processes of semantic information of constituent concepts. On the other hand, in the relational combination, there was no difference in RTs between the modifier and head probes. However, we did not find a different RT pattern between the combination and non-combination conditions, which needs additional interpretation. Since the mean RTs of both the modifier and head probes in relational combination were the fastest among all conditions in Experiment 2, this may reflect that these RTs were the ceiling point at which both probes were as activated as possible across all task conditions. Unless the semantic feature was not affected by additional inhibition as shown in the head probe during property combination in the combination task condition, the mean RTs would be the same with or without involving the combinatorial process during relational combination. Future research may be needed to examine this ceiling effect in more detail.

## General Discussion

We found that when people interpret novel property compounds, such as “feather luggage” as “a light luggage,” they replace a specific semantic feature of the head noun (“heavy”) with that of the modifier noun (“light”). On the other hand, when people interpret novel relational compounds, such as “piano blanket” as “a blanket that covers the piano,” they complete the semantic feature of the head noun (“cover”) with the whole modifier concept (“piano”). Across two experiments, we examined these two differential combinatorial processes by measuring the asymmetric involvement of semantic information in modifier and head nouns immediately after combination processes. Our findings consistently showed asymmetric activation patterns of semantic features in property and relational combinations. Specifically, we found that the intrinsic features of the modifier concepts were more activated in property combination, while the intrinsic features of the head concepts were inhibited. Furthermore, we found that these activation patterns only occurred after the combinatorial process was involved as semantic priming, indicating that property combination involves replacing the semantic feature of the head noun with that of the modifier noun. Finally, we found that in relational combination there was no difference in the level of semantic activation between the extrinsic feature of the head noun and the whole concept of the modifier nouns, indicating that relational combination involves completing the extrinsic feature of the head noun with the entire modifier concept rather than replacing one with another. These novel findings provide significant insight into the fundamental mechanisms of human creativity and suggest that a specific type of semantic information determines how concepts are combined.

Although feature-based models demonstrate that conceptual combination is a process of systematic modification of the specific slot of the head noun either with specific properties of the modifier concept as in property combination or with the whole modifier concept as in relational combination (Murphy, 1988, 1990; Wisniewski, 1996; Wisniewski, 1997; Wisniewski and Love, 1998; Gagné, 2000; Choi and Shin, 2010), they have not specified how these processes engage with differential semantic features. Using a semantic priming paradigm, our study directly examined whether different types of semantic features are activated when individuals interpret property and relational combinations. We provided converging evidence that property and relational combinatorial processes involve different semantic features and that those semantic features elicit asymmetric courses and mechanisms of semantic integration. Immediately after interpreting property combination, the semantic activation of intrinsic features of the modifier noun was greater than that of the same dimension of the head noun. This implies that the intrinsic semantic feature of the modifier noun replaces the specific value of the head noun. On the other hand, immediately after interpreting the relational combination, the extrinsic semantic feature of the head noun and the whole modifier did not show different levels of activation. This indicates that the semantic information from both the modifier and head nouns, particularly the extrinsic feature of the head noun and the entire modifier concept, is needed to complete relational combination rather than replacing one with another.

A potential alternative explanation might be that the different semantic activations between property and relational combinations could be driven by the different lexical-semantic information not by different combinatorial mechanisms. We created noun–noun compounds that could be easily interpreted as property or relational combinations. For property compounds, the modifier noun had a salient intrinsic feature, and the head noun shared the same feature dimension as the intrinsic feature of the modifier noun. On the other hand, for the relational compounds, the head noun had a salient extrinsic feature, and the modifier could be an object of the verb for the salient extrinsic feature of the head noun. As such, the different activation on the LDT could be attributed to the distinctive lexical-semantic characteristics of property and relational compounds. However, we did not find different activation patterns between the property and relational combinations when participants processed the same semantic information but did not combine the two noun concepts (i.e., the non-combination task condition in Experiment 2). This finding further supports that activation of differential semantic information is solely due to the different combinatorial processes of property and relational combinations rather than to the lexical-semantic processes of each constituent concept.

Recent studies in neuroscience on conceptual combination have further increased the need to functionally define the semantic combinatorial process. For example, studies on the neural substrates of conceptual combination have shown that semantic integration involves activation of the AG (Boylan et al., 2015, 2017; Price et al., 2015, 2016), ATL (Baron et al., 2010; Baron and Osherson, 2011; Bemis and Pylkkänen, 2011, 2013; Coutanche and Thompson-Schill, 2015; Boylan et al., 2017), and right-hemisphere temporoparietal regions (Graves et al., 2010). However, further research is needed because it remains elusive what this activation means in terms of the mechanisms that each combinatorial process entails. To understand the implications of neural activation more precisely, the necessity to specify the process in more detail from the beginning to the end of conceptual combination is increasing. Moreover, previous research has been limited from discerning the neural substrates of property and relational combinations. Of the few studies that have specifically examined the neural substrates of each combination, Boylan et al. (2017) showed that property and relational combinations differentially engage two semantic hubs of the brain; while the AG demonstrated greater activation for relational combination, the left ATL showed an earlier peak for property combination. However, this finding does not address how the neural difference indicates a functional difference.

The ability to group the two concepts together instantly, whether taxonomically or thematically, will help to elucidate how humans deal with meanings in general and the overall character of human thinking. Although it has been widely accepted that conceptual combination requires creative thinking, the relationship between them remains unspecified. Among a few studies that have assessed this relationship, Wan and Chiu (2002) showed that engaging in conceptual combination can enhance creativity including divergent thinking (DT) as measured by Torrance Tests of Creative Thinking (Torrance, 1974). However, relatively little research has investigated beyond the correlation between task performance for DT or convergent thinking (CT) and that for conceptual combination. As conceptual combination has been advocated as an essential component of creativity, research questions including “How can conceptual combination be defined from a perspective of creative thinking?” and “How is it underpinned with dynamic interactions between different brain systems?” should be delineated.

Previous findings from creativity research have revealed that creative cognition involves two distinct subprocesses: idea generation and idea evaluation. Idea generation refers to stimulating spontaneous self-generative process (i.e., DT), while idea evaluation is tantamount to the effortful goal-directed control over self-generated ideas (i.e., CT). The dual-pathway model of creativity also points out this duality of spontaneous and controlled processes in creative thinking (Nijstad et al., 2010). It is interesting to find the parallels between property vs. relational combinations and controlled vs. spontaneous processes in creative thinking. From a dual-pathway model of creativity’s point of view, relational combination corresponds to the spontaneous pathway since the pathway suggests stimulating creativity through a flexible switching between multiple categories or concepts. The relational combination can be facilitated if the information *outside* the constituent concepts supports the potential thematic relation between the constituent concepts. For example, “piano blanket” can be better understood as “a blanket that covers piano” if other concepts, such as “dust” or “protection,” are provided as informative clues. On the other hand, the property combination corresponds to the controlled pathway since the pathway leads to creativity through a rather in-depth exploration inside just a few categories. Property combination can be facilitated when information *inside* the constituent concepts is thoroughly explored. For example, “feather luggage” can be understood as “light luggage” without any supplementary information as in the relational combination.

In contrast to their antagonistic relationship, creative cognition has been shown to involve both goal-directed and self-generated thought processes. Recent neuroscientific research further corroborates this framework that creative cognition involves dynamic interaction of the default and control brain networks that correspond to spontaneous idea generation and controlled idea evaluation, respectively (Beaty et al., 2016; Khalil et al., 2019). Conceptual combination is also expected to involve these two components of creative cognition. However, how and the extent to which both processes are engaged in conceptual combination have not been specified. Noun–noun conceptual combination starts with spontaneous semantic memory retrieval of constituent concepts and ends with accommodating compatible and incompatible features to construct a congruent semantic structure. Somewhere between these two ends, divergent thinking in which potential interpretations are generated and convergent thinking in which the plausibility of generated meanings is evaluated would be implemented. Although a few studies have suggested conceptual combination taps creative problem solving rather than divergent creativity (Kohn et al., 2011), whether interpretation of novel compounds also follows the order of idea generation and then idea evaluation remains unclear. Although generating multiple potential interpretations and then selecting best one among them are possible, generating a single interpretation that best satisfies the combination from the beginning is also possible. Moreover, a single framework cannot explain all conceptual combination tasks, including interpretation generation, sensicality judgment, and exemplar generation, since the demands of task-specific goals vary.

As future research, the process of conceptual combination should be elaborated from the perspective of creative thinking, specifically DT and CT processes. Delineating brain network dynamics, especially default and executive control networks during conceptual combination, would also be informative. Obtaining such insights are expected to benefit creativity education by facilitating and training students to be more creative. Methodological variations including computational neuroscientific methods will also be of interest in future work to determine the specific algorithmic process and differences between the two combinatorial types (Costello and Keane, 1997; Lynott and Ramscar, 2001; Perlovsky and Levine, 2012; Khalil et al., 2019; Günther et al., 2020).

Although our findings demonstrate asymmetric mechanisms between property and relational combinations, the present work has several limitations. First, we attempted to investigate the processes underlying property and relational combination by examining activation patterns immediately after completing combinatorial processes. Even though our approach has a closer temporal proximity between combinatorial processes and the measure of semantic activation (i.e., LDT) than the traditional interpretation paradigm, we did not capture semantic activations in the middle of semantic integration. Future research may need to test semantic activation patterns during combinatorial processes by manipulating stimulus onset asynchrony between the presentation of noun–noun compounds and the subsequent LDT.

Second, the present study used interpretation bias data as a proxy for whether the property or relational combinatorial process was primed instead of examining how a specific property or relational compound word was actually interpreted. This could be one caveat since our study did not directly measure how each trial of a property or relational compound was interpreted to determine whether the subsequent LDT was influenced by property combinatorial processing or relational combinatorial processing. Instead, it focused on the average effect at the group level. However, as shown in Table 1, even though the pretest result showed high interpretation convergence (over 90% of property compounds were interpreted in the property combination, and over 90% of relational compounds were interpreted in the relational combination), there were still variabilities in interpretations (72–100% for property compounds and 67–100% for relational compounds). As our main interest was to measure the activated semantic features immediately after processing a given noun–noun compound word without any interruption, we did not measure verbal interpretations immediately after the sensicality task, which could have confounded the subsequent LDT RT. However, the direct measurement of how each participant interprets each compound would be more ideal since for some of the compounds, there is no guarantee that all participants applied congruent combinatorial processing to compound types. Future research should explore better experimental methods or designs to demonstrate a more direct measurement of how specific combinatorial processing occurs and affects the subsequent LDT or other possible appropriate tasks.

Third, this study did not specify what effect the combinatorial process has on the features that are not directly relevant to the comprehension of a compound word. We only compared activation patterns for features that were expected to be utilized in each combination type (i.e., intrinsic features in property combination vs. extrinsic features and the whole concept in relational combination); thus, it is unclear how the two combinatorial processes utilize other features of the modifier and head nouns. In particular, for relational combination, the whole modifier noun is associated with an extrinsic feature of the head noun. We tested whether the entire modifier concept is activated by using a probe word that designates a primary part of the modifier concept (e.g., the “keyboard” of a “piano”), but it is unclear whether other features were also similarly activated (e.g., the “strings” or “pedals” of “piano”). Future research may be necessary to examine how property and relational combinations influence the semantic activation of other features that have a less essential role in conceptual combination to examine how far the influence of a semantic combination reaches in modifying the constituent concepts.

Fourth, we used a between-subject design for the combinatorial type in this study and this could be one caveat since this could develop different training effects per condition. The reason why we adopted a between-subject design was to better handle the difficulty of the task. Contrary to the relatively simpler tasks including lexical decision or categorization task that require subjects to access to lexical entries and activate its semantic information, conceptual combination further requires complex semantic coordination processes. Many results of interpretation generation task of novel compound words have well shown that they need at least more than 1,000 ms to successfully complete the semantic combination which is contrary to the lexical access of which response latency is generally under 500 ms. Since the focus of this study was to examine and compare the asymmetric characteristics of property and relational combinatorial processing and the main dependent variable was RT in a LDT that followed the interpretation of compound words, not comparing the interpretation latency of each compounds, we assumed that there was less solid ground to expect that the potential difference in practice effect between the combinatorial types would be directly transferred to the lexical decision RT. In this regard, we decided that alleviating the task difficulty would be the desirable control over using within-subject design for the compound type. From the relation-based point of view, it can be expected that property condition could be relatively easier to develop a task strategy than relational condition since in property combination, the subjects would be able to utilize only one relation which is “resemblance,” while in relational combination, the subjects should select the proper thematic relation among others, resulting in shorter comprehension latency in property combination than relational combination (we thank the anonymous reviewer for raising this point). To examine this possibility, we compared RT in the sensicality task between property and relational conditions. Contrary to expectation, we rather found a slightly longer RT in property combination than in relational, but the difference was not significant (in Study 1: *M*_relation_ = 1.73 s, *SD* = 0.70 vs. *M*_property_ = 1.83 s, *SD* = 0.72; *t* = 0.94, *p* = 0.348; in Study 2: *M*_relation_ = 1.38 s, *SD* = 0.71 vs. *M*_property_ = 1.63 s, *SD* = 1.64; *t* = 1.56, *p* = 0.120). Although we did not observe the selective training effect in this study, the between-subject design can be a caveat, and applying a better experimental design will be of interest in future work.

Fifth, the two experiments in this study showed high RT exclusion rates (37% in Experiment 1; 51% in Experiment 2). These percentages reflect composite values of dropped RTs due to failing to meet the five selection criteria. Breaking down each component, the exclusions based on RT outliers were 7% in Experiment 1 and 14% in Experiment 2. The rest of the exclusions was from failed combinatorial processes from respondents (i.e., ‘no’ responses in the sensicality task). One possibility of the high exclusion rates might be because the experiments were conducted online. Online experiments enable researchers to investigate their research ideas with a more general population. However, since online experiment settings are less controlled than laboratory experiments, participants in online experiments could be distracted or affected by other uncontrolled factors than in laboratory experiments. Thus, future research may need to test the current research findings by systematically controlling outliers (e.g., implementing a sufficient number of practice trials, providing incentives to increase task engagement, or setting a time window that participants can submit their answers; we thank the anonymous reviewer and the editor for raising this point).

One final point worth mentioning is that although this study inherits schema-modification theory, which takes a feature-based approach, this study focused on delineating the characteristics of semantic combinatorial processing mechanisms by leveraging the prediction that different types of semantic features asymmetrically influence the processing of property and relational combinations rather than on seeking to determine specific perspective over another. This is contrary to the previous research that focused on evaluating claims made by the dual-process theory, which is a representative of the property-based model (Wisniewski and Love, 1998; Estes, 2003), and the CARIN theory, which is a representative of the relation-based model (Gagné, 2000), to determine which perspective to take for examining whether there is a distinctive underlying process for each property and relational comprehension. This study aimed to be one of the preliminary attempts to explore the influence of different types of semantic information, whereby asymmetric combinatorial processing may entail. Even though previous noun–noun conceptual combination studies have well defined the characteristics of each property and relational combination, most previous studies are heavily based on verbal outputs (i.e., generated interpretations) rather than focusing on the underlying online processes of both combination types. However, we found it more important to precisely elucidate the detailed course and mechanism of semantic combinatorial processing, determining which type of semantic information is involved and what course of operation is applied to the information, since elaborating the processing-level account on how people combine concepts may provide a richer basis to examine which approach among attributive or relational approach would better represent conceptual combination. For instance, Gagné and Spalding (2007) showed similar results to this study, demonstrating that the process of conceptual combination can affect the availability of features in the head noun’s representation. They showed that semantic features that are incompatible with the meaning of the compound word temporarily become less available during subsequent processing of the head noun, which is directly related to the results of property combination in this study. Even though Gagné and Spalding’s study and this study take different approaches, feature-based and relation-based, respectively, and they did not differentiate combinatorial types in their work, it is interesting that both studies identify the similar phenomenon. It is expected that future research could provide a richer basis to determine which approach can better explain conceptual combination by deepening the understanding of each combinatorial process.

In conclusion, we have shown in two experiments that property and relational combinations entail distinct processes of semantic integration involving differential semantic information and combinatorial mechanisms. We found that in property combination, the semantic activation of intrinsic features in modifier was greater than the semantic features of the same dimension in the head, whereas in relational combination, the extrinsic semantic features in the head and the entire modifier showed equal activation, and these patterns of semantic activation only occurred when the combinatorial process was involved, unlike when the same lexical-semantic information was accessed, yet the integration process was not engaged. These convergent findings indicate that the property combination is a process of replacing the semantic features of the head with the modifier’s intrinsic features, whereas the relational combination is a process of relating the head’s extrinsic semantic features with the entire modifier concept demonstrating two asymmetric ways of integrating semantic information.

## Data Availability Statement

Data available at: https://osf.io/t5p8a/.

## Ethics Statement

The studies involving human participants were reviewed and approved by the University of Dayton Institutional Review Board. All participants provided their written informed consent to participate in this study.

## Author Contributions

MC designed research and drafted the manuscript. MC and SY collaboratively performed the research and analyzed the data. All authors provided critical revisions and approved the final version of the manuscript.

## Conflict of Interest

The authors declare that the research was conducted in the absence of any commercial or financial relationships that could be construed as a potential conflict of interest.

## Publisher’s Note

All claims expressed in this article are solely those of the authors and do not necessarily represent those of their affiliated organizations, or those of the publisher, the editors and the reviewers. Any product that may be evaluated in this article, or claim that may be made by its manufacturer, is not guaranteed or endorsed by the publisher.
